# Dual Pro- and Anti-Inflammatory Features of Monocyte-Derived Dendritic Cells

**DOI:** 10.3389/fimmu.2020.00438

**Published:** 2020-03-27

**Authors:** Waqas Azeem, Ragnhild Maukon Bakke, Silke Appel, Anne Margrete Øyan, Karl-Henning Kalland

**Affiliations:** ^1^Department of Microbiology, Haukeland University Hospital, Helse Bergen, Bergen, Norway; ^2^Department of Clinical Science, University of Bergen, Bergen, Norway; ^3^Broegelmann Research Laboratory, University of Bergen, Bergen, Norway; ^4^Department of Immunology and Transfusion Medicine, Haukeland University Hospital, Helse Bergen, Bergen, Norway; ^5^Norway Centre for Cancer Biomarkers, University of Bergen, Bergen, Norway

**Keywords:** monocyte-derived dendritic cell, pro-inflammatory, tolerogenic, immunotherapy, beta-catenin

## Abstract

The transcription factor β-catenin is able to induce tolerogenic/anti-inflammatory features in different types of dendritic cells (DCs). Monocyte-derived dendritic cells (moDCs) have been widely used in dendritic cell-based cancer therapy, but so far with limited clinical efficacy. We wanted to investigate the hypothesis that aberrant differentiation or induction of dual pro- and anti-inflammatory features may be β-catenin dependent in moDCs. β-catenin was detectable in both immature and lipopolysaccharide (LPS)-stimulated DCs. The β-catenin inhibitor ICG-001 dose-dependently increased the pro-inflammatory signature cytokine IL-12p70 and decreased the anti-inflammatory signature molecule IL-10. The β-catenin activator 6-bromoindirubin-3′-oxime (6-BIO) dose-dependently increased total and nuclear β-catenin, and this was associated with decreased IL-12p70, increased IL-10, and reduced surface expression of activation markers, such as CD80 and CD86, and increased expression of inhibitory markers, such as PD-L1. 6-BIO and ICG-001 competed dose-dependently regarding these features. Genome-wide mRNA expression analyses further underscored the dual development of pro- and anti-inflammatory features of LPS-matured moDCs and suggest a role for β-catenin inhibition in production of more potent therapeutic moDCs.

## Introduction

Dendritic cells (DCs) are the most efficient antigen-presenting cells of the immune system and play a vital role in initiating the adaptive immune response and maintaining tolerance to self-antigens (1). Immature DCs continually search their environment for antigens, while mature DCs migrate to the lymph nodes and present processed antigens on their major histocompatibility (MHC) molecules to T cells. Traditionally, three different types of DCs have been considered in peripheral blood, plasmacytoid DCs (pDC) and classical or conventional DC type 1 (cDC1) and type 2 (cDC2) (2–4). Recent high-resolution technologies have revealed additional types of human blood DCs and progenitors (5, 6). Altogether, DCs comprise less than 1% of circulating blood leukocytes, and for this reason, most DC-based therapies have relied on DCs generated *in vitro* from the more plentiful blood monocytes [reviewed in (2–4, 7, 8)]. Monocyte-derived DCs (moDCs) are able to activate the immune system, but it may be anticipated that there is a considerable potential for the generation of more potent and robust DCs for cancer therapy.

Depending on their phenotype and type of secreted cytokines, DCs may exert either pro-inflammatory or tolerogenic function as their response to newly encountered antigens. The transcription factor β-catenin can be activated to stimulate tolerogenic features of DCs, such as cytokine, surface marker, and metabolic profiles (9–12). Surface markers associated with pro-inflammatory activation include CD80 and CD86, whereas PD-L1 and PD-L2 are considered inhibitory or tolerogenic markers (7). Interleukin 12 (IL-12p70) represents a pro-inflammatory cytokine (13) and interleukin 10 (IL-10) an anti-inflammatory or tolerogenic cytokine (14) that can be secreted from mature DCs.

Inhibiting β-catenin signaling could have a dual effect in cancer therapy, as this pathway promotes tolerogenic features of the local dendritic cells and is often activated in cancer and cancer stem cells. In the present study, β-catenin activation was achieved using a specific inhibitor of the β-catenin destruction complex, 6-bromoindirubin-3′-oxime (6-BIO) that has been found to increase β-catenin in the cell nucleus of different cell types (15). In this way the central, final part of β-catenin signaling downstream of the destruction complex can be investigated. This approach has experimental advantages because β-catenin activation is impacted by different up-stream pathways with complicated cross-talks (16). Likewise, central β-catenin inhibition was attempted using the small molecule ICG-001 that binds CREB-binding protein (CBP) to disrupt its interaction with β-catenin and inhibits CBP function as a co-activator of β-catenin-mediated transcription at regulatory genomic elements (17).

In the present study, moDCs derived from buffy coats of healthy donors were investigated and revealed the potential of mature moDCs to co-develop both pro-inflammatory and tolerogenic features assayed by IL-12p70 and IL-10 secretion, DC surface markers, and whole-genome mRNA quantification.

## Materials and Methods

### Generation of Monocyte-Derived Dendritic Cells

Buffy coats of healthy blood donors at the Blood bank of Haukeland University Hospital, Bergen, were used to generate human monocyte-derived dendritic cells (moDCs). Informed consents were obtained from all donors, and samples were anonymized according to the approval by the Regional Ethical Committee (#64205). Healthy donors were above 23 years of age. Peripheral blood mononuclear cells (PBMCs) were isolated by gradient centrifugation using Lymphoprep™ (Cat. No. 1114545; Axis-Shield). Pan Monocyte Isolation Kit (Cat. No. 130-096-537; MiltenyiBiotec) with the addition of CD61 MicroBeads (Cat. No. 130-051-101; MiltenyiBiotec) and LS columns (Cat. No. 130-042-401; MiltenyiBiotec) were used to separate untouched monocytes from PBMCs by indirect magnetic labeling. Monocytes were then cultured in CellGenix GMP DC medium (Cat. No. 20801-0500; CellGenix) supplemented with 20 ng/ml of IL-4 (Cat. No. 11340047; Immunotools) and 100 ng/ml of GM-CSF (Cat. No. 11343128; Immunotools) at cell densities of 1.5 × 10^6^ per 3 ml/well in six-well plates or 0.75 × 10^6^ per 1.5 ml/well in 12-well plates for 4 days. IL-4 and GM-CSF were replenished on day 3. The fourth-day cultures were treated with compounds at different concentrations, i.e., 6-bromoindirubin-3-oxime (6-BIO; Cat. No. S7198; Selleckchem) 1 nM to 2 μM and/or ICG-001 (Cat. No. S2662; Selleckchem) 0.5 to 8 μM (for 24 h), and 1 h later with 30 ng/ml of LPS (for 23 h). As controls, the vehicle DMSO was added in LPS-treated and un-treated (iDC) populations. The moDCs were harvested on day 5.

### Western Blots

Western blots were performed as previously described (18). moDCs were lysed in RIPA-buffer (Cat. No. ab156034; Abcam) supplemented with 1:100 Protease Inhibitor Cocktail Set I (Cat. No. 535142; Calbiochem). The protein concentration was quantified using Direct Detect^®^ Infrared Spectrometer (EMD Millipore) using Direct Detect^®^ Assay-free Cards (Cat. No. DDAC00010-GR; Millipore). Twenty micrograms was used for each sample loaded onto BoltTM Bis-Tris Plus Gels (Cat. No. NW04120BOX; Novex; Life Technologies). The proteins were separated by SDS electrophoresis and blotted on Amersham Hybond P 0.45 PVDF blotting membrane (Cat. No. 10600069; GE Healthcare). The primary antibodies used were anti-β-catenin (Cat. No. 16051; Abcam) and anti-GAPDH (Cat. No. MAI-16757 Invitrogen). The horseradish peroxidase (HRP)-conjugated secondary antibodies used were anti-rabbit (dilution 1:2,000; Cat. No. NA934; Amersham) and anti-mouse (dilution 1:2,000; Cat. No. 170-501; Bio-RAD). SuperSignal™ West Pico PLUS Chemiluminescent Substrate (Cat. No. 34580; Thermo Scientific) was used for visualization with Chemidoc XRS, and images were captured using Quantity One 4.6.5 software (Bio-Rad). MagicMark™ XP Western Protein Standard (Cat. No. LC5602; Invitrogen) was used as molecular weight marker. ImageJ 1.50i software (National Institutes of Health, Bethesda, MD, USA) was used to quantify the band intensity of each protein followed by normalization to its corresponding GAPDH control.

### Indirect Immunofluorescence Assay

Indirect immunofluorescence assays were performed as previously described (19). The primary antibody used was anti-β-catenin (Cat. No. 16051; Abcam) at 1 μg/ml dilution, and FITC-conjugated Pierce™ goat anti-rabbit IgG (H + L) secondary antibody (Cat. No. 31635; Thermo Scientific) was used at 1:50 dilution. Cells grown on coverslips were mounted on glass slides in SlowFade™ Gold Antifade Mountant w/DAPI (Cat. No. S36939; Invitrogen). The images were captured on Leica TCS SP8 STED 3× confocal microscope using Leica Application Suite X 2.0.2.15022 software (Leica Microsystems).

### Enzyme-Linked Immunosorbent Assay (ELISA)

Secretion of IL-12p70 and IL-10 in the supernatant of moDC cultures were measured using IL-12p70 Human Uncoated ELISA Kit (Cat. No. 88-7126-88; Invitrogen) and IL-10 Human Uncoated ELISA Kit (Cat. No. 88-7106-88; Invitrogen), respectively. The absorbance was measured with Synergy H1 Hybrid Multi-Mode Reader and analyzed using Gen5 2.00.18 software (BioTek). Data are presented as fold change to DMSO-treated control sample, as absolute levels of IL-10 and IL-12 secretion varied considerably between the donors.

### Flow Cytometry

The phenotype of the generated moDC populations was determined by flow cytometry as described previously (20). In short, 1 × 10^5^ moDCs were incubated with FcR-blocking reagent (Cat. No. 130-059-901; MiltenyiBiotec) before titrated amounts of a panel of nine antibodies were added for 10 min at room temperature in the dark. The antibodies used were as follows: CD83 PE-CF594 (Cat. No. 562631; BD Biosciences), HLA-DR Horizon V500 (Cat. No. 561224; BD Biosciences), CD80 Brilliant Violet 605 (Cat. No. 305225; Biolegend), CCR7 Brilliant Violet 421 (Cat. No. 353208; Biolegend), CD86 Alexa Fluor 647 (Cat. No. 305416; Biolegend), CD274 PE-Cyanine7 (Cat. No. 46-5983-42; eBioscience), CD273 PerCP-Fluor 710 (Cat. No. 46-5888-42), CD14 FITC (Cat. No. 21620143; Immunotools), and CD1a PE (Cat. No. 21270014; Immunotools). The cells were analyzed on LSR Fortessa (BD Biosciences), and further analysis was performed using FlowJo V10 software (FlowJo, LLC). Unstained samples were used to set the gates, and 1% false-positive events were accepted throughout the analysis. For each experiment, a minimum of 5,000 single events were recorded.

### Luminex Microbead Cytokine Assay

The cell-free supernatants collected from the MLR cultures were thawed and measured for cytokines using Human Magnetic 25-Plex Panel (Cat. No. LHC009M; Invitrogen) according to the manufacturer's instructions. We measured and analyzed seven cytokines including interferon-α (IFN-α), interferon-γ (IFN-γ), tumor necrosis factor-α (TNF-α), interleukin (IL)-6, IL-2R, IL-10, and IL-12. Luminex plates were read using the Luminex 100 System (Luminex Corporation, Austin, TX, USA) following the manufacturer's instructions. STarStation V3.0 (Build: 3810.0; Applied Cytometry Systems, Sheffield, UK) was used to analyze the data.

### Mixed Leukocyte Reaction (MLR)

Allogeneic MLR was performed as previously described (20). In short, 2 × 10^5^ monocyte-depleted allogeneic PBMCs were labeled with CFSE using Vybrant™ CFDA SE Cell Tracer Kit (Cat. No. V12883; Invitrogen) and co-cultured with 5 × 10^4^ moDCs of different donors for 5 days in X-Vivo 20 medium (Cat. No. 04-448Q; Lonza) supplemented with 50 U/ml of IL-2 (Cat. No. 11340023; Immunotools) and 10 ng/ml of IL-7 (Cat. No. 11340073; Immunotools). The cells were harvested and analyzed on Accuri C6 flow cytometer (BD Biosciences). For each experiment, a minimum of 20,000 single events were recorded.

### DNA Microarray Analyses

Genome-wide transcription profiling using Agilent microarrays has been described previously (21). Total RNA was isolated and tested for RNA integrity by 1% agarose gel electrophoresis, then converted to Cy3-labeled cRNA targets and hybridized to Agilent Whole Human Genome 44k Microarrays (Cat. No. G4845A; Agilent Technologies). Raw data were imported and analyzed in J-Express software (http://www.molmine.com) (22). We used mean spot signals as intensity measure, normalized the expression data over the entire arrays, and log2-transformed and considered genes changed more than 1.5-fold with FDR value <5% as differentially expressed genes. DNA microarray data have been deposited into the ArrayExpress database under accession number E-MTAB-8330.

### RNA Sequencing and Analyses

All experiments were conducted at QIAGEN Genomic Services. The library preparation was done using TruSeq^®^ Stranded mRNA Sample preparation kit (Illumina Inc.). The starting material (500 ng) of total RNA was mRNA enriched using the oligodT bead system. The isolated mRNA was subsequently enzymatically fragmented. Then first-strand synthesis and second-strand synthesis were performed, and the double-stranded cDNA was purified (AMPure XP, Beckman Coulter). The cDNA was end repaired, 3′ adenylated and Illumina sequencing adaptors ligated onto the fragments ends, and the library was purified (AMPure XP). The mRNA stranded libraries were pre-amplified with PCR and purified (AMPure XP). The libraries' size distribution was validated and quality inspected on a Bioanalyzer 2100 or BioAnalyzer 4200tape Station (Agilent Technologies). High-quality libraries were pooled based in equimolar concentrations based on the Bioanalyzer Smear Analysis tool (Agilent Technologies). The library pool(s) were quantified using qPCR, and the optimal concentration of the library pool was used to generate the clusters on the surface of a flow cell before sequencing on a NextSeq 500 instrument (75 cycles) according to the manufacturer instructions (Illumina Inc.).

### Software Tools Used for RNA-Seq Analysis

NGS data analysis pipeline was based on the Tuxedo software package, which is a combination of open-source software, and implements peer-reviewed statistical methods. In addition, specialized software developed internally at QIAGEN Genomic Services was employed to interpret and improve the readability of the final results. The components of NGS data analysis pipeline for RNA-seq include Bowtie2 (v. 2.2.2), see (23), Tophat (v2.0.11), see (24, 25), and Cufflinks (v2.2.1), see (26, 27).

To guide the assembly process, an existing transcript annotation was used (RABT assembly). In addition, fragment bias correction was used to correct for sequence bias during library preparation (28). When comparing groups, Cuffdiff was used to calculate the FPKM (number of fragments per kilobase of transcript per million mapped fragments) and test for differential expression.

### Statistical Analysis

All data were analyzed using GraphPad Prism 8 (GraphPad software). Statistical significance of the difference was calculated using one-way analysis of variance (ANOVA) with Dunnett's multiple comparisons test or two-way ANOVA with Tukey's multiple comparisons test, and 95% confidence interval. A value of p ≤ 0.05 was considered statistically significant.

## Results

### β-Catenin Accumulation in Monocyte-Derived Dendritic Cells

**Figure 1:**
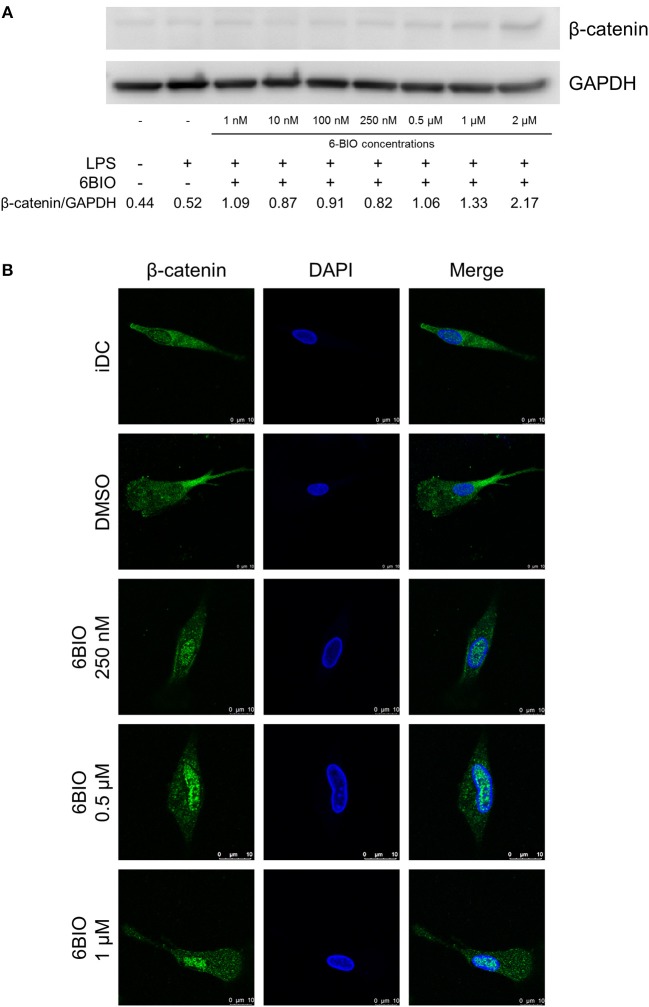
Monocyte-derived dendritic cells (moDCs) obtained from healthy donors were treated with indicated concentrations of 6-bromoindirubin-3′-oxime (6-BIO) or DMSO (vehicle) for 24 h together with 30 ng/ml of lipopolysaccharide (LPS) for the last 23 h or left untreated as iDC. **(A)** β-catenin protein levels were measured by Western blot in whole-cell lysates. Representative Western blot of three independent experiments is shown. **(B)** Fluorescein (FITC) indirect immunofluorescent detection of β-catenin proteins according to confocal microscopy analyses.

β-catenin was detectable in immature moDCs (iDCs) by Western blotting, and this concentration increased slightly in moDCs matured for 23 h using LPS (Figure 1A). 6-BIO dose-dependently increased the accumulation of β-catenin when moDCs were treated with 6-BIO for 24 h and with concomitant LPS for the last 23 h prior to cell harvesting according to Western blot analyses (Figure 1A). Indirect fluorescent confocal microscopy revealed β-catenin accumulation in the cell nuclei of moDCs treated with 0.25 to 1 μM 6-BIO (Figure 1B and Supplementary Figure 1).

### 6-BIO Dose-Dependently Promoted Anti-Inflammatory Features of moDCs

**Figure 2:**
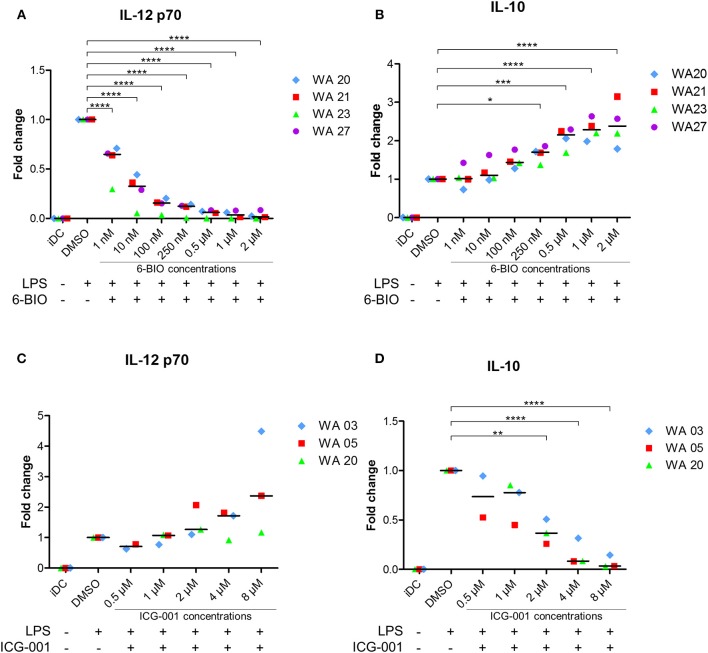
moDCs obtained from healthy donors were treated with indicated concentrations of 6-BIO or ICG-001 or DMSO for 24 h with 30 ng/ml of LPS for the last 23 h or left untreated with those compounds as iDC. **(A–D)** The fold changes compared to DMSO controls of IL-12p70 and IL-10 in supernatants were measured by enzyme-linked immunosorbent assay (ELISA). Each symbol represents a different donor, and lines represent the median. *p ≤ 0.05, **p ≤ 0.01, ***p ≤ 0.001, ****p ≤ 0.0001 using one-way ANOVA with Dunnett's multiple comparisons test and 95% confidence interval.

In order to further investigate possible immune-relevant consequences of 6-BIO-induced β-catenin in mature moDCs, we quantified IL-12p70 and IL-10 of cell culture supernatants using ELISA. As exemplified in Figure 2A, the IL-12 concentration decreased significantly with increasing 6-BIO concentrations following LPS maturation for 23 h. 6-BIO concentrations down to 1 nM decreased IL-12 levels compared to cultures with only vehicle and with pronounced dose-dependent IL-12 decrease at 10, 100, and 250 nM. On the contrary, IL-10 secretion increased dose dependently and significantly with increasing 6-BIO in LPS-matured moDCs (Figure 2B). Absolute levels of IL-10 and IL-12 secretion varied considerably in moDCs of buffy coats donated by different healthy persons, although the above trends were in common, for which reason fold change was used for the Y-axis of Figure 2. Quantitative levels in picogram/milliliter are shown in Supplementary Figure 2.

### ICG-001 Increased IL-12 and Decreased IL-10 Secretion of Mature moDCs

In order to examine the possibility that β-catenin signaling was activated in LPS-matured moDCs even without the use of 6-BIO, cells were treated with the commercially available β-catenin inhibitor ICG-001 for 24 h and with LPS added for the last 23 h before harvest of supernatants. ICG-001 between 1 and 8 μM dose-dependently increased the secretion of IL-12p70 (Figure 2C). In the same supernatants, a dose-dependent decrease in IL-10 was found with significantly increasing effect from 1 to 8 μM ICG-001 (Figure 2D).

### 6-BIO and ICG-001 Competed Against Each Other Regarding Pro- and Anti-Inflammatory Features

**Figure 3:**
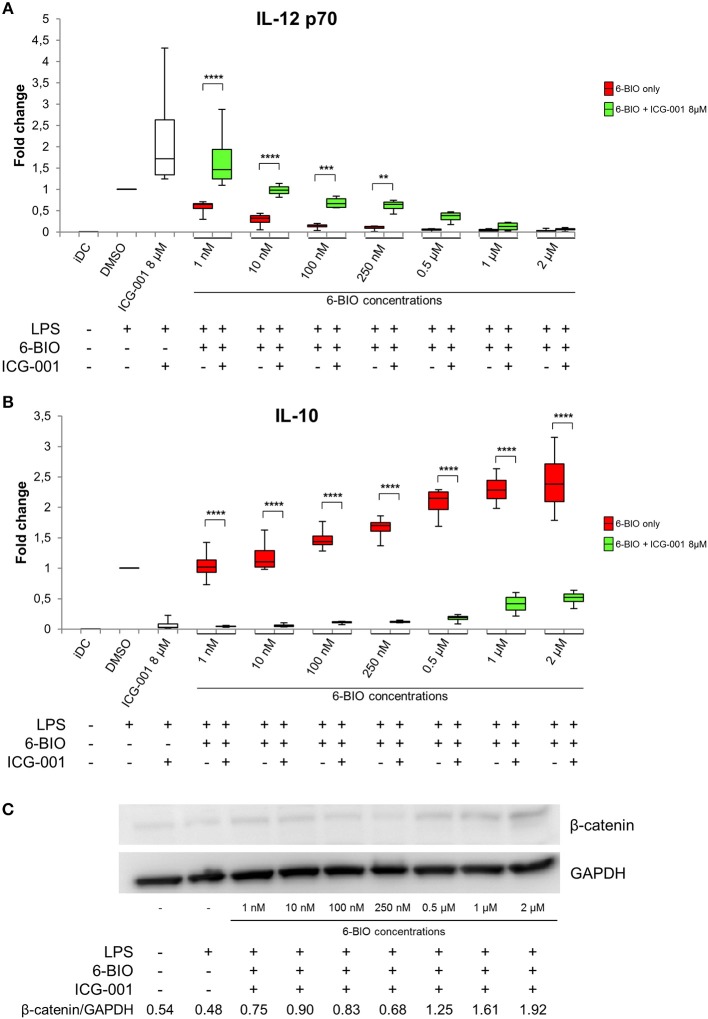
moDCs obtained from healthy donors were treated with 8 μM ICG-001 and/or indicated concentrations of 6-BIO or DMSO (vehicle) for 24 h with 30 ng/ml of LPS for the last 23 h or left untreated as iDC. **(A,B)** Each vertical box and whisker plot shows the fold change compared to untreated DMSO controls of IL-12p70 and IL-10, respectively, measured by ELISA. The lines represent the median, edges of the box represent 25th and 75th percentiles, and whiskers display the smallest and highest value, *n* = 5. **(C)** β-catenin protein levels were measured by Western blot in whole-cell lysates. *p ≤ 0.05, **p ≤ 0.01, ***p ≤ 0.001, ****p ≤ 0.0001 using two-way ANOVA with Tukey's multiple comparisons test and 95% confidence interval.

To examine any direct competition between 6-BIO and ICG-001, ELISA was used to quantify IL-12 and IL-10 secretion of LPS-matured DCs (Figure 3). IL-12 induced by either LPS alone or by LPS plus 8 μM ICG-001 was dose-dependently competed by 6-BIO (Figure 3A). Correspondingly, IL-10 induced by either LPS alone or by LPS plus 6-BIO was efficiently competed by 8 μM ICG-001 (Figure 3B). Western blots showed that ICG-001 did not significantly affect the 6-BIO-induced accumulation of β-catenin (Figure 3C). Quantification of mRNA levels using Agilent microarray and RNA-seq profiling showed that β-catenin mRNA levels remained relatively unaffected by ICG-001 treatment and was reduced by possible negative feedback following 6-BIO treatment (Supplementary Figure 3).

### Effect of 6-BIO and ICG-001 on Maturation and Activation Markers of DCs

**Figure 4:**
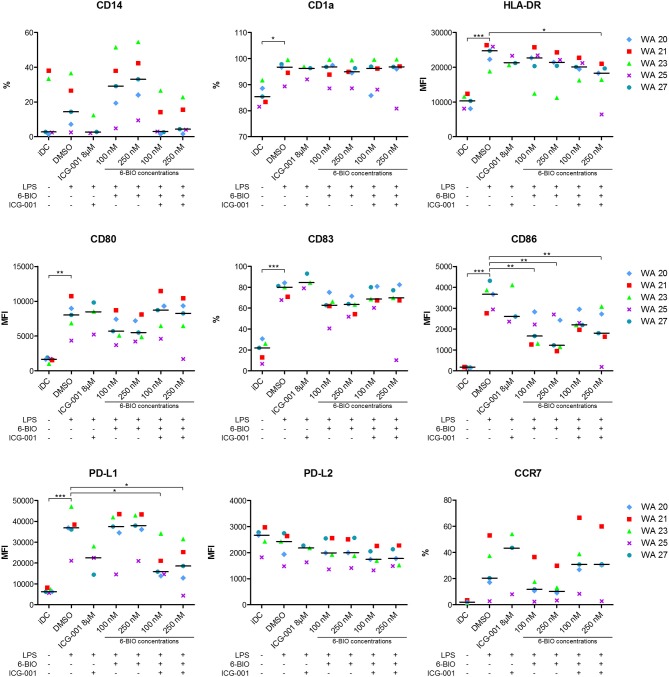
moDCs obtained from healthy donors were treated with 8 μM ICG-001 and/or indicated concentrations of 6-BIO or DMSO for 24 h with 30 ng/ml of LPS for the last 23 h or left untreated as iDC. Flow cytometry analyses of cell surface markers were performed. Each symbol represents a different donor and lines represent the median. *P ≤ 0.05, **P ≤ 0.01, ***P ≤ 0.001, using one-way ANOVA with Dunnett's multiple comparisons test and 95% confidence interval. %, percentage of positive cells; MFI, median fluorescence intensity.

Flow cytometry was used to examine how relevant DC surface markers were affected by β-catenin stimulation or inhibition. A representative gating strategy is shown in Supplementary Figure 4. Retained monocyte marker CD14, along with decreased CD1a, suggests deviated maturation of DCs (29). ICG-001 treatment decreased the percentage of LPS-matured DCs that expressed CD14. 6-BIO (100 and 250 nM) increased the CD14 expression, and this was counteracted by the addition of 8 μM ICG-001 (Figure 4). CD1a expression was not affected by either treatment. The maturation markers HLA-DR and CD83 showed, as expected, a clear upregulation following LPS treatment for 23 h. HLA-DR was not strongly affected by either ICG-001 or 6-BIO. 6-BIO was moderately inhibitory and ICG-001 moderately stimulatory to CD83 expression at the concentrations tested (Figure 4).

The activation markers CD80 and CD86 increased strongly as expected when iDCs were stimulated for 23 h with LPS. 6-BIO clearly reduced expression of LPS-induced CD80 and CD86, while the effect of ICG-001 was less clear (Figure 4). The inhibitory marker PD-L1 increased strongly following LPS maturation of iDC for 23 h with or without 6-BIO. ICG-001 (8 μM) tended to decrease the LPS-induced PD-L1 with or without 100 or 250 nM 6-BIO. The PD-L2 marker was not increased by LPS, but was moderately decreased by 8 μM ICG-001 (Figure 4). The DC migration marker CCR7 was upregulated during LPS maturation, as expected, and was clearly reduced by 6-BIO treatment. ICG-001 increased CCR7 expression and competed the 6-BIO-associated downregulation of CCR7.

### Effect of 6-BIO and ICG-001 on Gene Expression Patterns

**Figure 5:**
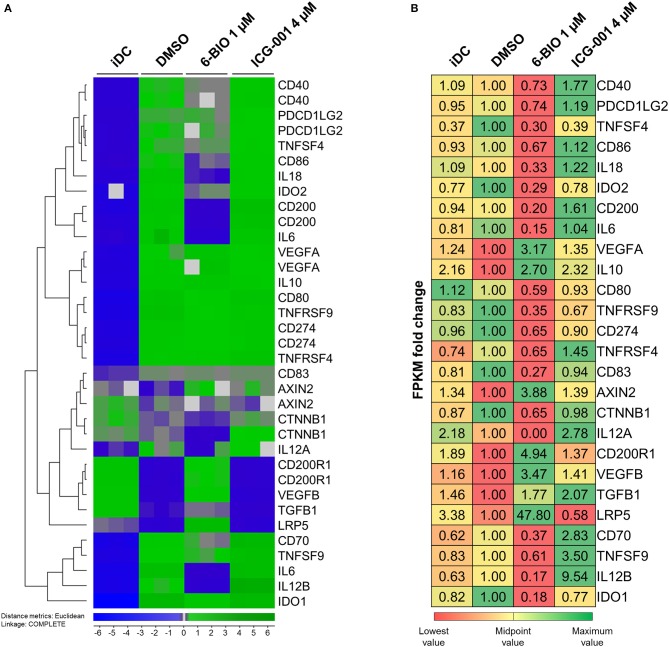
moDCs obtained from healthy donor and treated with DMSO, 1 μM 6-BIO or 8 μM ICG-001 for 24 h with 30 ng/ml of LPS for the last 23 h, or left untreated as iDC. **(A)** Total RNA was subjected to whole-genome microarray analysis. Heat map of selected gene expression data based on the supervised hierarchical cluster analysis (J-Express™ software) of different treatments. **(B)** Total RNA (500 ng) was used to generate RNA-seq libraries. Values of the expression of selected genes were based on fold change of normalized fragments per kilobase of transcript per million mapped fragments (FPKM) for each gene of each sample. Fold change was compared to untreated DMSO (vehicle) controls.

In order to obtain a broader overview of potential pro- and anti-inflammatory features of LPS-matured moDCs, mRNA transcription was profiled using both Agilent 44k microarrays and Illumina RNA-seq. Figure 5 shows a selection of 26 genes known to be relevant in pro- and anti-inflammatory regulation. The performance of the model with gene expression analyses was exemplified by the known β-catenin target gene *LRP5* (30). Both Agilent 44k microarrays and Illumina RNA-seq showed a significant, but low level, iDC expression of *LRP5* mRNA, with almost 48-fold induction of *LRP5* in LPS-matured moDCs treated with 6-BIO. In contrast, *LRP5* was reduced in ICG-001-treated LPS-matured moDCs. The classical β-catenin target gene *AXIN2* was relatively weakly expressed as mRNA, but with significant induction with concomitant 6-BIO in LPS-matured moDCs (Figures 5A,B). *CTNNB1* (β-catenin) mRNA was relatively abundant and was reduced by 6-BIO and little affected by ICG-001 in LPS-matured moDCs.

### Transcription Levels of Pro- and Anti-Inflammatory Interleukins

According to Agilent 44k microarray data (Figure 5A) pro-inflammatory interleukins, *IL-6, IL-12B*, and *IL-18* were strongly induced in LPS-matured moDCs compared to their immature origins. RNA-seq data validated the increase in *IL-6* and *IL-12B* (Figure 5B). Parallel treatment with 1 μM 6-BIO clearly reduced mRNA levels of all of *IL-6, IL-12B, IL18*, and *IL-12A* according to both microarray and RNA-seq analyses. ICG-001 (4 μM) induced the opposite effect and a clear increase in these pro-inflammatory mRNA markers in LPS-matured moDCs according to both data sets. The anti-inflammatory *IL-10* mRNA was increased in LPS-matured moDCs compared to iDCs according to microarray data, but this was not validated by RNA-seq data. Concomitant 6-BIO-treatment, however, was associated with increased *IL-10* mRNA according to both data sets with less clear effect of concomitant ICG-001 treatment.

### Transcription Levels of Activating and Inhibitory Membrane Markers

The DC activation markers *CD40, CD80*, and *CD86* were clearly increased in LPS-matured moDCs according to microarrays (Figure 5A). These results were validated by RNA-seq data for *CD86*, but not for *CD40* and *CD80*. Concomitant 6-BIO and LPS treatment showed consistently lower levels of all these cell surface activation markers according to microarrays in comparison with only LPS and, in particular, with concomitant LPS and ICG-001 treatment. RNA-seq data validated the 6-BIO results for *CD40, CD80*, and *CD86* and showed clearly higher expression levels when ICG-001 was used instead of 6-BIO in LPS-matured moDCs (Figure 5B). DC membrane inhibitory markers *PDCD1LG2* (PD-L1) and *CD274* (PD-L2) mRNAs were induced in LPS-matured moDCs according to both data sets, while concomitant 6-BIO treatment reduced transcription and ICG-001 increased transcription according to RNA-seq data. *TNFS4* (OX40 ligand) is typically expressed on DCs and its receptor *TNFRSF4* (OX40) typically on T lymphocytes (31). According to microarray data, *TNFRSF4* increased substantially in LPS-matured DCs and was reduced by concomitant 6-BIO and increased by ICG-001 according to both microarrays and RNA-seq (Figures 5A,B). *TNFSF9* (4-1BBL; CD137L) expressed on DCs activate lymphocytes via binding to *TNFRSF9* (CD137) (32). Microarray data showed strongly induced expression of both *TNFSF9* and *TNFRSF9* mRNAs in LPS-matured moDCs. Concomitant 6-BIO treatment was associated with lower, and ICG-001 treatment with higher, *TNFSF9* expression (Figures 5A,B). CD70 is a cell-membrane-bound TNF superfamily (TNFSR) member that activates T lymphocytes via TNFRSF member CD27 (31). According to microarray data, *CD70* mRNA was strongly induced by LPS maturation of moDCs, and this was reduced by 6-BIO and increased by ICG-001 (Figures 5A,B). CD200 is a surface glycoprotein that can induce IDO expression following engagement of pDC-expressed CD200R1. According to microarray data, *CD200* mRNA was strongly induced in LPS-matured moDCs. According to both microarray and RNA-seq data, *CD200* expression is further increased by concomitant ICG-001, but reduced by concomitant 6-BIO (Figures 5A,B). *CD200R1* was clearly reduced in LPS-matured moDCs, but was increased by concomitant 6-BIO treatment (Figures 5A,B).

### Transcription Levels of Inhibitory Enzymes and Secreted Proteins

Indoleamine 2,3-dioxygenase (IDO-1) activity, via enzymatic catalysis of tryptophan metabolites, converts mature DCs into tolerogenic antigen-presenting cells that suppress T effector cells and promote T regulatory cells, thereby promoting tolerance (33). *IDO1* was found strongly upregulated in LPS-stimulated moDCs. Concomitant 6-BIO reduced *IDO1* (Figures 5A,B). TGFB is a secreted immunomodulatory molecule of DCs (10, 31). According to both microarray and RNA-seq data, *TGFB1* is reduced in LPS-stimulated moDCs compared to iDCs. Concomitant 6-BIO treatment stimulated *TGFB1* expression (Figures 5A,B). VEGF expression and secretion are associated both with aberrant DC maturation and anti-inflammatory DCs (34), and both *VEGFA* and *VEGFB* increased strongly in LPS- plus 6-BIO-treated moDCs (Figures 5A,B).

### Functional Effects of 6-BIO and ICG-001 in the Allogeneic Mixed Leukocyte Reaction (MLR)

**Figure 6:**
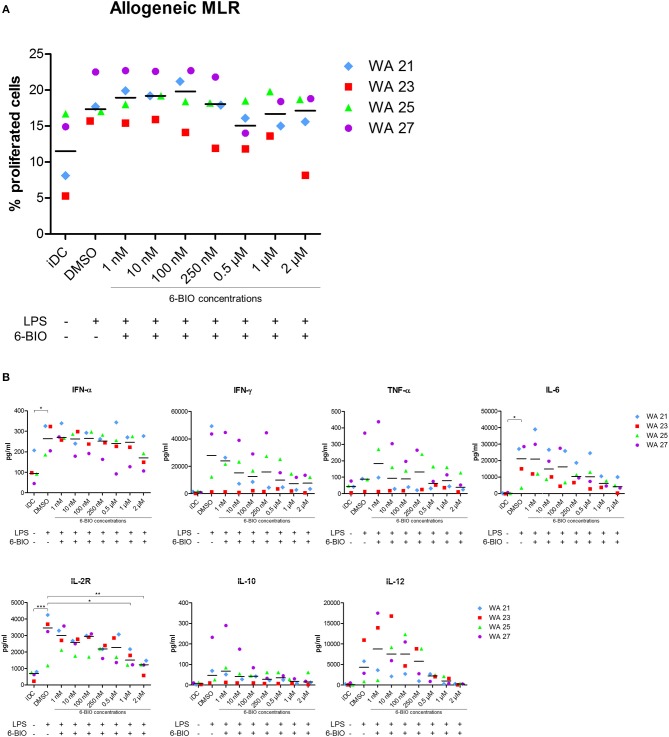
Mixed leukocyte reaction (MLR) between moDCs and allogeneic peripheral blood mononuclear cells (PBMCs) obtained from healthy donors. moDCs were treated with indicated concentrations of 6-BIO or DMSO for 24 h with 30 ng/ml of LPS treatment for 23 h, or left untreated as iDC. Allogeneic PBMCs were pre-labeled with CFSE and co-cultured with treated moDCs for 5 days. **(A)** Percentage proliferated cells was measured by reduction in CFSE intensity. **(B)** The amount of secreted cytokines was analyzed using 25-Plex Panel, and the concentrations are given in pg/ml. Each symbol represents a different donor, and lines represent the median. *p ≤ 0.05, **p ≤ 0.01, ***p ≤ 0.001, using one-way ANOVA with Dunnett's multiple comparisons test and 95% confidence interval.

Allogeneic MLRs were performed to analyze T-cell stimulatory capacity of the generated DC populations. The cells were co-cultured with monocyte-depleted allogeneic CFDA-SE-stained PBMCs, and proliferation was determined by the reduction in CFSE intensity. All DC populations showed T-cell stimulatory capacity with LPS-matured moDCs inducing more proliferation than iDC (Figure 6A). The addition of 6-BIO did not, however, show any significant variation in T-cell proliferation compared to DMSO control, although a slight decrease was observed at 0.5 and 2 μM 6-BIO. Neither did up to 8 μM ICG-001 significantly affect the recorded T-cell proliferation (result not shown). In order to examine further the apparent minimal effect of β-catenin modulation of the MLR assay, co-culture supernatants were investigated for cytokines using the Luminex microbead assay (Figure 6B). There was a trend toward 6-BIO dose-dependent reduction in pro-inflammatory cytokines IFN-γ, TNF-α, and IL-6. Among those cytokines, only IL-6 level reached statistical significance. Soluble IL-2R showed a statistically significant and dose-dependent decrease with increasing 6-BIO. It was additionally noted that the 6-BIO dose-dependent effects on IL-10 and IL-12 that we found in pure DC cultures seemed to be abrogated in co-cultures with allogeneic lymphocytes.

## Discussion

Improved DCs are much needed for next-generation cancer immunotherapy (2, 7, 8, 35). One attractive possibility is that β-catenin signaling can be exploited to generate more robust and potent therapeutic DCs. The transcription factor β-catenin is an important immune regulator that can affect pro-inflammatory and anti-inflammatory/tolerogenic features of mouse and human DCs of different subtypes (11, 12, 36–40), but the therapeutic potential of β-catenin inhibition in moDCs needs further clarification (41–47).

In the present work, we established a model system of moDCs derived from buffy coats of healthy blood donors. Monocytes were induced to become immature DCs (iDCs) using GM-CSF and IL-4 for 4 days. Thereafter, small molecular compounds were added to iDCs for 24 h with concomitant LPS for the last 23 hours, followed by assays of secreted IL-10 and IL-12p70 and surface markers of maturation, activation, and inhibition.

In order to investigate the possibility that a basal β-catenin activation was present in LPS-matured moDCs (48), even in the absence of 6-BIO, we tested our model with the β-catenin inhibitor ICG-001, previously found to inhibit β-catenin-stimulated transcription in both cancer cells (49) and dendritic cells (42). A pronounced ICG-001 dose-dependent increase in IL-12p70 and a corresponding dose-dependent decrease in IL-10 secretion was observed when iDCs were treated for 24 h with ICG-001. This suggested that β-catenin is activated in mature moDCs to promote anti-inflammatory features and, furthermore, is accessible to β-catenin inhibition, even in the absence of Wnt ligands or other specific activators of Wnt or β-catenin signaling added to the culture medium.

Addition of the small molecular compound 6-BIO indicated, however, that the basal β-catenin activity of LPS-matured moDCs can be augmented by external stimulation. Western blot analyses of moDC whole-cell lysates detected β-catenin both in iDCs and LPS-matured moDCs with dose-dependent increase in β-catenin accumulation detectable from low 6-BIO concentrations. Confocal microscopy revealed obvious nuclear accumulation of β-catenin at 250 nM and higher concentrations of 6-BIO. This *in situ* localization method was preferred over nuclear and cytoplasmic fractionation, consistent with our previous results that fractionation is associated with loss of labile nuclear proteins due to the drop of colloid osmotic pressure during fractionation (50).

The small molecule ICG-001 binds CREB-binding protein (CBP) to disrupt its interaction with β-catenin and inhibits CBP function as a co-activator of Wnt/β-catenin-mediated transcription (17). This mechanism of β-catenin inhibition is consistent with our present findings. According to our Western blot assays, ICG-001 did not affect β-catenin at the protein synthesis/stability level. According to microarray and RNA-seq data, β-catenin (*CTNNB1*) mRNA levels were similar in ICG-treated and non-treated moDCs. ICG-001 was consequently used to examine further the association between β-catenin inhibition and anti-inflammatory features of mature moDCs. Competition assays between 6-BIO and ICG-001 showed that these compounds had inverse effects on IL-10 and IL-12p70 secretion and on dendritic cell markers of activation, inhibition, and migration. 6-BIO dose-dependently promoted anti-inflammatory patterns of the examined cytokines and several surface markers. ICG-001 exhibited the opposite and the competing effect on cytokine and several surface markers. The pronounced upregulation of the β-catenin target gene *LRP5* in 6-BIO-treated and downregulation in ICG-001-treated LPS-matured moDCs additionally support the existence of a basal and dynamic β-catenin activation status.

IL-10 is considered an anti-inflammatory cytokine that is induced by β-catenin signaling (11) and by non-canonical Wnt5a signaling in mouse DCs (39). Stimulation of toll-like-receptors (TLR2, TLR4, TLR5, TLR7, and TLR9) has been shown to induce IL-10 production of DCs [reviewed in (14)]. The TLR4 stimulator LPS induced dual production of IL-10 and IL-12 in human moDCs, but with significant donor-to-donor variation (13, 51), consistent with our present findings. In the LPS-matured moDCs, the level of β-catenin was dose-dependently increased by 6-BIO. It may offer experimental advantages to activate β-catenin downstream of the cytoplasmic destruction complex because several upstream and parallel pathways, such as WNTs, tankyrases (52, 53), and TLR2 stimulation via PI3 kinase and ERK in moDCs (54, 55) may activate nuclear β-catenin signaling. In parallel with increasing β-catenin levels, 6-BIO dose-dependently increased IL-10 and dose-dependently decreased IL-12p70. These experiments, therefore, reveal the potential of β-catenin signaling to modulate important pro- and anti-inflammatory cytokine production of moDCs. The IL-10 promoter has been shown to contain several response elements that can bind β-catenin to activate IL-10 transcription (56). Both microarray and RNA-seq data showed increased IL-10 mRNA in 6-BIO-treated moDCs. The relationship between β-catenin signaling and IL-12 stimulation is additionally complex due to the ability of IL-10 to decrease IL-12 production in DCs (13).

In mice, DC-specific deletion of the Wnt co-receptors LRP5/6 or β-catenin led to an increased expression of IL-6, TNF-α, IL-1β, IL-12p40, and IL-12p70 with diminished production of IL-10 and TGF-β (57). In another study on mouse splenic DC precursors, CD11c-specific constitutive β-catenin activation upregulated Irf8 through targeting of the Irf8 promoter, β-catenin-stabilized CD8a + DCs secreted elevated IL-12 upon *in vitro* microbial stimulation, and pharmacological β-catenin inhibition using ICG-001 blocked this response in wild-type cells (42). Also, knock-down of the non-canonical Wnt5a in human moDCs, presumed not to signal via β-catenin, compromised IL-12 secretion (58).

In order to obtain an additional impression of potential pro- and anti-inflammatory features of LPS-matured moDCs, whole-genome mRNA analyses of moDCs was done, using both Agilent microarray and Illumina RNA sequence quantifications. Recently, it was published that β-catenin directly stimulates the *IDO1* transcription and additionally the peroxisome proliferator-activated receptor-γ (PPAR-γ), thus, causing the metabolic shift required for protoporphyrin X synthesis, the heme prosthetic group required for full IDO1 enzymatic activity in DCs (12). According to our mRNA expression data, *IDO1* was strongly induced and abundantly expressed in LPS-matured moDCs, while *PPARG* was relatively abundantly expressed in both immature and LPS-matured DCs (ArrayExpress E-MTAB-8330). Surprisingly, 6-BIO dose-dependently reduced *IDO1* mRNA levels in our LPS-matured moDCs, although significant *IDO1* mRNA was still expressed even at high 6-BIO concentrations. Interestingly, CD200 binding to CD200R1 on murine pDCs has been reported among stimulators of IDO1 expression (59). Expressions of *CD200* and *CD200R1* were strongly affected during LPS maturation and by concomitant 6-BIO and ICG-001 treatment. These findings have to be followed up separately.

It has been shown in both murine and human models that Wnt5a from melanomas affects the local DCs to express indoleamine 2,3-dioxygenase-1 (IDO1) that stimulates development of T regulatory cells (TRegs) through kynurenine (60). Wnt5a has been considered to be a ligand of the non-canonical Wnt pathway (β-catenin independent), but appears to be able to context-dependent stimulation of β-catenin signaling, in some cases stronger than the “canonical” Wnt3a stimulation (12, 60).

TGF-β induces T regulatory cells and thereby promotes tolerance when secreted from antigen-presenting cells [(10, 31, 48) and references therein]. It has been reported that TGF-β antagonizes β-catenin in DCs, thereby selectively suppressing signaling associated with tolerogenic DC activation while having no impact on LPS-induced, β-catenin-independent immunogenic activation (61). According to our microarray and RNA-seq data, *TGFB1* was clearly reduced during LPS-mediated maturation of moDCs, but this decrease was counteracted by both concomitant 6-BIO and ICG-001 treatment. This could represent indirect effects of β-catenin, also because *TGFB1* is not a known β-catenin target gene.

*VEGF* has been shown to be a direct β-catenin target gene in different cell types (62). The strong 6-BIO-enhanced expression of both *VEGFA* and *VEGFB* would suggest this to be the case in LPS-matured moDCs. VEGF has been shown to be immunosuppressive in different ways: it can inhibit the function of T cells, increase the recruitment of T regulatory cells and myeloid-derived suppressor cells (MDSCs), and hinder the differentiation and activation of DCs (34).

Additional transcriptional determinants of tolerogenic and immunogenic states during dendritic cell maturation have been published (63). All our genome-wide Agilent microarray and Illumina RNA-seq data have been made publicly available and can be further explored regarding β-catenin targets and pro- and anti-inflammatory transcription of LPS-matured moDCs.

Mixed leukocyte reaction (MLR) was employed for the assessment of T-cell stimulatory capacity of the generated cell populations and showed the ability of the LPS-stimulated moDCs to induce allogeneic T-cell proliferation. The minor effects of either 6-BIO or ICG-001 in the MLR assay could reflect that the outcome of conflicting pro- and anti-inflammatory cues could be complex. It is possible that negative feedback effects due to moDC and allogeneic cross-talk could result in the abrogation of the pronounced dose-dependent effects on both IL-10 and IL-12 secretion by either 6-BIO or ICG-001 in pure moDC. Expanded cytokine profiling of MLR culture supernatants showed 6-BIO dose-dependent decreases of several relevant cytokines. Presently, this is a reminder of increased complexity once different immune cells are brought into interaction. Future work will address such interactions in MLR assays and in DC and patient-derived cell co-cultures with expanded assays including parallel multi-variable mass cytometric analyses of supernatants and cells.

Much understanding is lacking regarding β-catenin signaling in moDCs, although a critical role of β-catenin signaling in DC function and differentiation of pro- and anti-inflammatory features *in vivo* is already established [reviewed in (37, 46)]. In both freshly isolated and Flt3-stimulated CD11c+ DCs from mouse lymph nodes, the main conclusion was that Wnts upregulate immune suppressive cytokines (IL-10, VEGF, TGF-β) without inhibiting LPS-induced maturation and activation, thus allowing development of a mature tolerogenic phenotype (39). Minimal effects were detected on the MHCII maturation marker or CD80 or CD86 activation markers or the migration marker CCR7 (39). IL-12p70 secretion was additionally found to be little affected by either canonical (Wnt3a) or non-canonical (Wnt5a) signaling in that study (39). In another study, however, deletion of β-catenin in a mouse model was found to increase expression of DC co-stimulatory markers (CD40, CD80, CD86) and to decrease the inhibitory markers PD-L1 and PD-L2 (64). Different murine tumor models have documented the ability of Wnt ligands to stimulate DCs to produce tolerogenic factors, such as IL-10, Raldh, and Ido-1 [reviewed in (11)]. In one study of human cancer, melanoma-intrinsic β-catenin signaling was found to inhibit DC migration and lead to immune evasion (65). The important mechanisms involved have been reviewed (38, 40, 66, 67).

## Conclusion

LPS-matured moDCs co-developed pro- and anti-inflammatory surface and secretory markers with considerable quantitative person-to-person variation. A basal β-catenin activation was present in LPS-matured moDCs and could be boosted dose dependently by the β-catenin activator 6-BIO and counter-acted dose-dependently by the β-catenin transcription complex inhibitor ICG-001 with inverse effects on IL-10 and IL-12 secretion. These observations should be taken into consideration for the production of more potent and robust therapeutic DCs.

## Data Availability Statement

DNA microarray data have been deposited into the ArrayExpress database under accession number E-MTAB-8330.

## Author Contributions

WA, RB, and AØ did the experiments. WA prepared the figures. All authors contributed to experimental design, evaluation of results, manuscript revisions, and approved the submitted version.

### Conflict of Interest

AØ and K-HK own stocks in the Company Alden Cancer Therapy II AS that has sponsored and will sponsor dendritic cell-based cancer immunotherapy, including the trial registered at ClinicalTrials.gov Identifier: NCT02423928. AØ and K-HK additionally are co-inventors of the United States Patent Application No. 15/771496 (US-2018-0344715) regarding novel beta-catenin inhibitor compounds for cancer therapy. None of these compounds have been used or addressed in the present manuscript. The remaining authors declare that the research was conducted in the absence of any commercial or financial relationships that could be construed as a potential conflict of interest.
