# Supplementary material for: Dual Pro- and Anti-Inflammatory Features of Monocyte-Derived Dendritic Cells
Source: Front Immunol. 2020 Mar 27;11:438. doi: 10.3389/fimmu.2020.00438 (PMC7120039; doi:10.3389/fimmu.2020.00438)
Supplement: Supplementary file 1 [file Data_Sheet_1.PDF]

Supplementary Materials

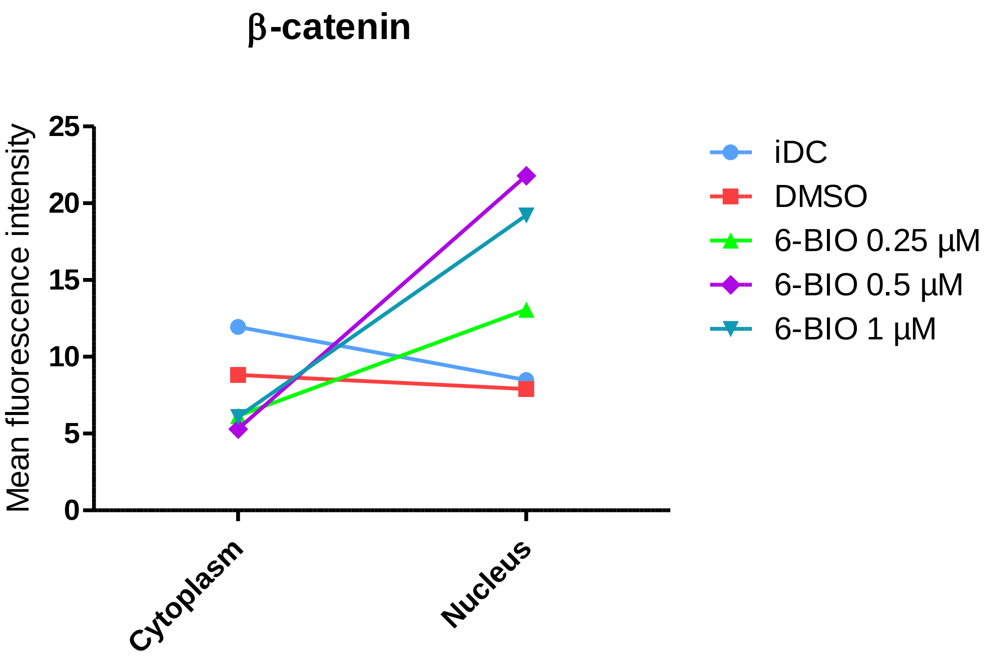

Supplementary Figure 1: The quantification of the mean fluorescence intensity of β-catenin signals in cytoplasm and nucleus are presented.
All fluorescence images were analyzed with ImageJ 1.52a software (National Institutes of Health, Bethesda, MD, USA).

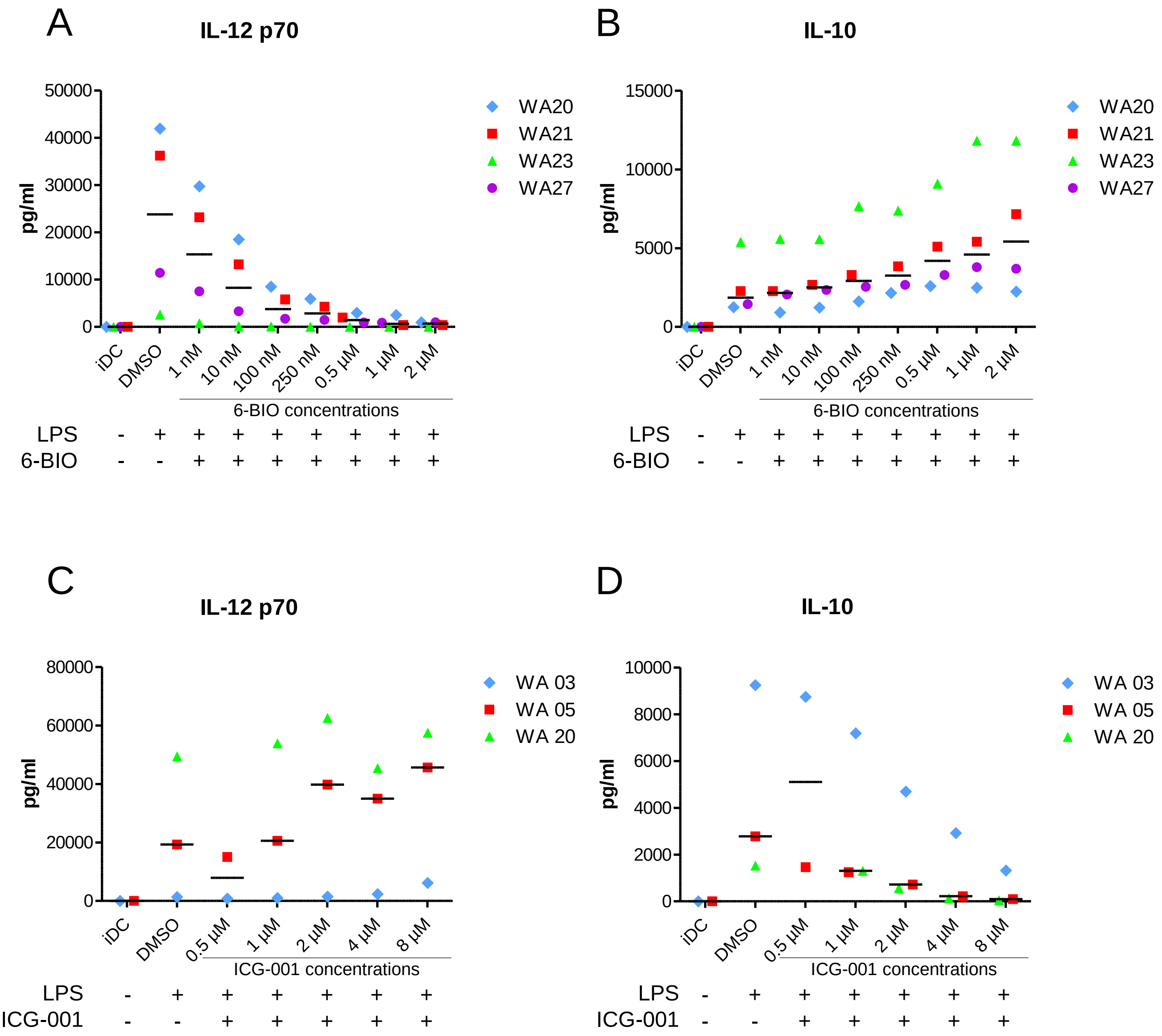

Supplementary Figure 2: moDCs obtained from healthy donors were treated with indicated concentrations of 6-BIO or ICG-001 or DMSO for 24 hours with 30 ng/ml LPS for the last 23 hours, or left untreated with those compounds as iDC. A) - D) The fold changes compared to DMSO controls of IL-12p70 and IL-10 in supernatants were measured by ELISA. Each symbol represents a different donor and lines represent the median.

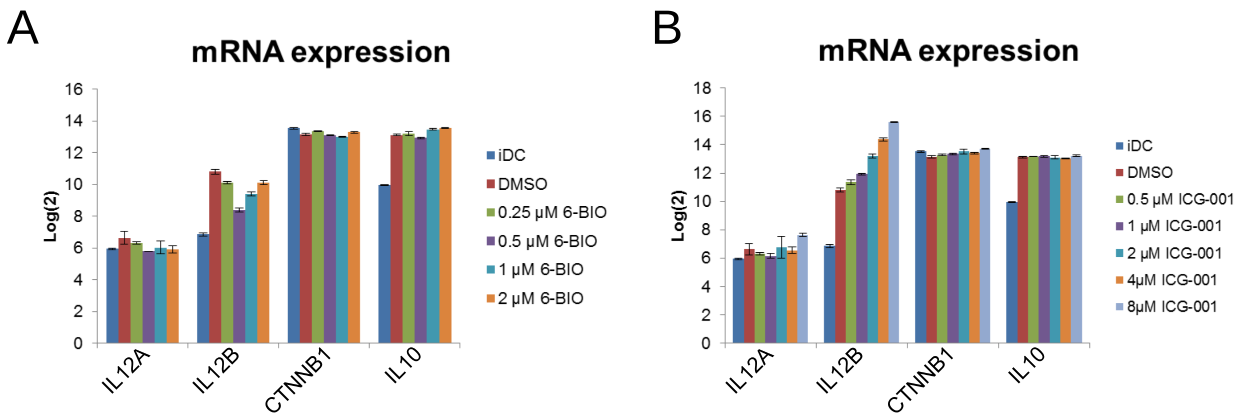

Supplementary Figure 3: moDCs obtained from healthy donors were treated different concentrations of 6-BIO or ICG-001 (as indicated) for 24 hours and corresponding controls were treated with DMSO followed by LPS 30 ng/ml treatment for 23 hours or left untreated as iDC. A) and B) Total RNA was subjected to whole-genome microarray analysis. Each colour represents a different treatment and error bars represent the standard deviation of the mean expression level.

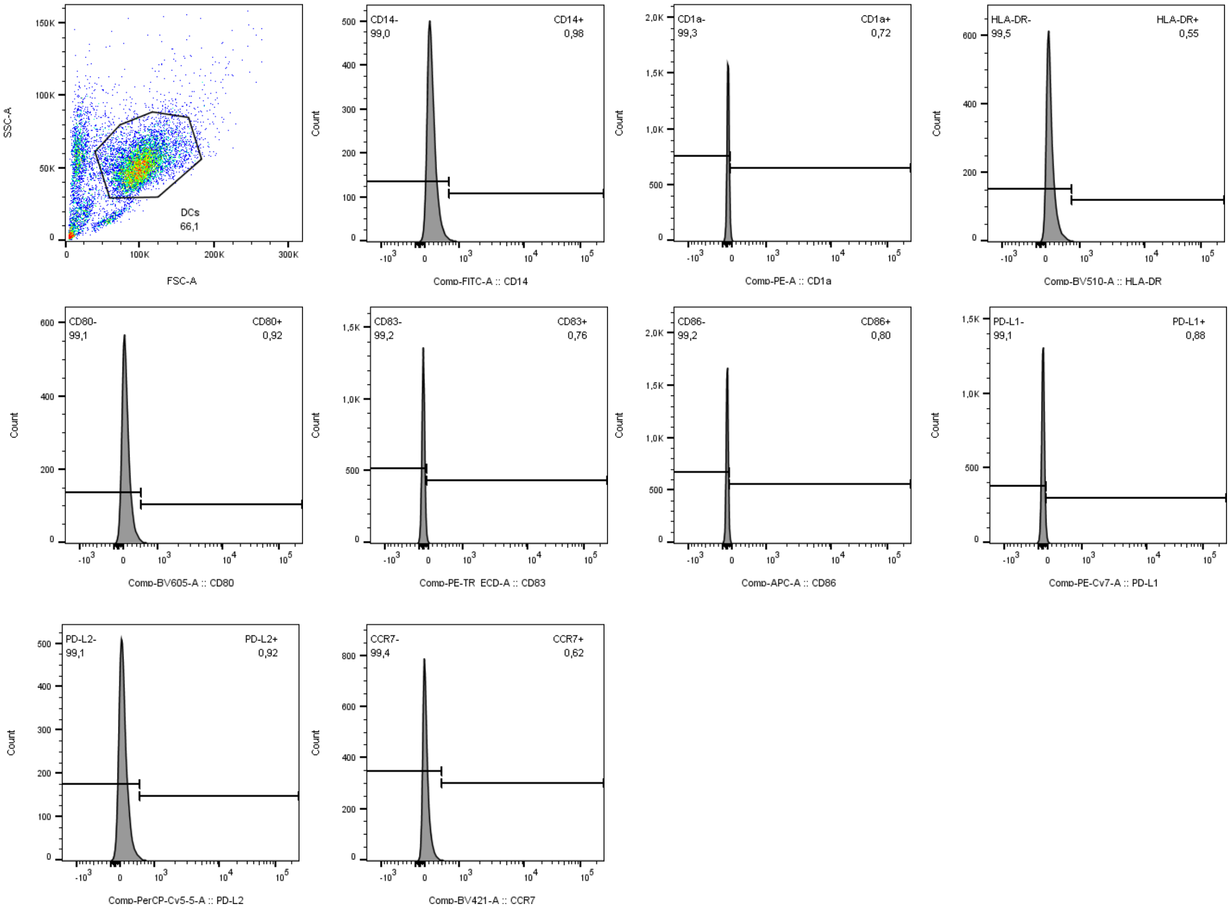

Supplementary Figure 4: Representative gating strategy for flow cytometry analysis of moDCs surface markers.
